# Practical Notes on Diagnosis and Treatment

**Published:** 1910-09-17

**Authors:** 


### Practical Notes on Diagnosis and Treatment.

**Examination of the Breast.**

Do not put your hand on the breast first; inspect it well before touching it. A tumour often causes a local flattening or an alteration of outline, so you may *see* a patient has cancer even more certainly than you can feel it. Always see both breasts and look for difference in level of the nipples, as well as any puckering or change in colour of the skin.—*Mr. Anthony Bowlby.*

**Chronic Mastitis and Cancer.**

Even if a mass should on microscopical examination prove to be inflammatory, it is wise to remove the chronically-inflamed portion of the breast, as this, if left, remains a constant menace of malignancy. When once a case comes under observation it should never be lost sight of until the inflammatory condition has entirely gone. It may be worth while trying *x*-rays before operation, only it must be borne in mind that if any thickened inflammatory material is left it is a source of danger.—*Mr. F. C. Wallis.*

**The Cure of Corns.**

There are few corns that will not succumb to a nightly bread poultice or water dressing, with a soft perforated plaster of spongiopilin by day to avoid friction. If the perforation can be filled with glycerin it will hasten the cure by keeping the hardened skin saturated by day as well as by night. Soaking the feet night and morning in warm water with the addition of some alkaline carbonate will, in many cases, remove corns.—*Dr. Foster Palmer.*

**Neuralgia.**

The method of deep parenchymatous injection of a very dilute solution of morphia, lodged at several places in the trunk of the affected nerve or around its sheath, is a combination of acu-puncture, aquapuncture, hypodermic injection, and anodyne medication. It may be safely tried for a few times at the beginning of the treatment; it is not only palliative, but sometimes a speedy and permanent curative effect follows, especially in neuralgic sciatica.—*Sir Wm. Whitla.*

